# Condylar Osteochondroma in an Older Adult With Facial Asymmetry: Case Report and Differentiation From Condylar Hyperplasia

**DOI:** 10.1155/crid/4143126

**Published:** 2026-08-13

**Authors:** Hisashi Ozaki, Erika Miyamoto, Hiroki Mori, Yosuke Kobayashi, Yoshihiko Yokoe, Yuichiro Imai

**Affiliations:** ^1^ Department of Oral and Maxillofacial Surgery, Kyoto Oral Health Care Center, Rakuwakai Otowa Hospital, Kyoto-shi, Kyoto, Japan; ^2^ Kyoto Jaw Deformity Center, Rakuwakai Otowa Hospital, Kyoto-shi, Kyoto, Japan; ^3^ Department of Oral and Maxillofacial Surgery, Nara Medical University, Kashihara-shi, Nara, Japan, naramed-u.ac.jp

**Keywords:** case report, hyperplasia, mandibular condyle, osteochondroma, temporomandibular joint

## Abstract

Condylar osteochondroma is a rare condition that is more common among middle‐aged women. Differential diagnoses for condylar osteochondroma include benign or malignant tumors and hyperplastic lesions. In particular, the differentiation between condylar osteochondroma and condylar hyperplasia is difficult due to similar clinical and imaging findings. Here, we describe a rare case of condylar osteochondroma in an older adult woman and discuss the differentiation from condylar hyperplasia, along with the clinical course. The patient was a 63‐year‐old woman with main complaints of facial asymmetry and malocclusion. Based on various imaging findings, the clinical diagnosis was a benign bone tumor of the left mandibular condyle. Low condylectomy was performed, improving facial asymmetry and malocclusion. Hematoxylin and eosin staining of the resected specimen demonstrated that the basic structure of the mandibular condyle was generally preserved. The surface of the tumor contained fibrous, cartilaginous, and cancellous bone layers. Chondrocytes were small, with no apparent cartilage islands. Based on these findings, a definitive diagnosis of condylar osteochondroma was made. Currently, histological examination is considered the most useful method for differentiating between condylar osteochondroma and condylar hyperplasia.

## 1. Introduction

Osteochondroma is described as osteocartilaginous exostosis [1]. According to the classification of the World Health Organization, osteochondroma is defined as a cartilage‐capped protrusion on the surface of a bone, with a marrow cavity linked to underlying bone [2, 3]. As the most common tumor affecting skeletal bones, this entity comprises approximately 35%–50% of all benign bone tumors [4], but is rarely found in the jaw [5]. In the mandible, the coronoid process and condyle are most often affected [4]. The mean age of patients is 39.7 years and the peak age range is the fourth decade. Incidence is reportedly greater among females than among males [6]. Osteochondroma of the condyle often results in facial asymmetry, deformity, malocclusion, pain, and dysfunction in the temporomandibular joint (TMJ). Surgical excision in the form of condylectomy is the treatment of choice. Notably, malignant transformation has been observed in osteochondromas [7]. Differential diagnoses for condylar osteochondroma include benign or malignant tumors and hyperplastic mandibular pathology [8]. Among these diseases, mandibular condylar hyperplasia (CH) often displays similar clinical symptoms, clinical course, and imaging findings, representing an obstacle to diagnosis. Currently, the final diagnosis is based on histological examination, including immunohistochemical staining. We encountered a case of condylar osteochondroma in a 63‐year‐old woman. Although she presented with facial asymmetry and malocclusion, good results were achieved with surgery. We report the favorable outcome of this case and the process used to achieve differentiation from CH.

## 2. Case Presentation

A 63‐year‐old woman presented in November 2023 with main complaints of progressive facial asymmetry and malocclusion. Five years previously, she had started noticing a gradual change in occlusion that resulted in deviation of the mandible to the right. Until this presentation, she had not experienced any difficulty or crepitation‐like noises in opening the mouth. She reported no pain associated with mouth opening. On initial examination, the mandible showed deviation to the right (Figure 1A). The mandibular central incisors were deviated 8 mm to the right toward the maxillary central incisors (Figure 1B). On opening the mouth, the mandible deviated to the left and maximum mouth opening was 44 mm. Mobility of the left condyle was good. No swelling of the lateral aspect of the TMJ was apparent on visual inspection. The patient was in good health with no history of facial trauma. On radiographic examination, a bone‐like radiopaque mass appeared posterior to the left mandibular condyle and there were no findings suggestive of hemimandibular elongation, which are more related to the progressive mandibular lateral deviation (Figure 2). Computed tomography (CT) and three‐dimensional computed tomography (3D‐CT) revealed a well‐defined, bone‐like, opaque mass located around the mandibular condyle (Figure 3A–C). Bone sclerosis was progressing within the tumor, unlike within the mandibular condyle. It was believed that the mandible had deviated to the right and forward due to the bony mass developed in the posterior region of the mandibular condyle. Bone scintigraphy showed increased uptake of radioactive isotope into the left TMJ region (Figure 4A). Radioactive uptake in the condyle on the affected side was found to be 3.69 times greater than on the unaffected side (Figure 4B). Magnetic resonance imaging (MRI) disclosed a mass lesion comprising cortical bone at the periphery, connected to the cortical bone of the condyle and osseous‐like tissue, which differs from the bone tissue within the condyle (Figure 5). A cartilaginous cap, which would usually appear as signal hyperintensity on T2‐weighted imaging, was not evident. From these findings, the clinical diagnosis was bone tumor of the left mandibular condyle, including peripheral osteoma and osteochondroma or mandibular CH. Low condylectomy was performed under general anesthesia. The upper and lower joint compartments of the TMJ were accessed using a preauricular approach (Figure 6A). To ensure an adequate safety margin from the lesion, low condylectomy with ultrasonic cutting tools—that is, osteotomy performed below the mandibular condyle and above the condylar neck—was performed (Figure 6A,B). If osteotomy is performed within the normal bone tissue, remodeling of the resected area (condyle) can be expected. Since the length of the mandibular ramus is also preserved, functional problems (such as mandibular deviation on centric occlusion or during mouth opening) can be avoided. The articular disk appeared normal, with no adhesion between the tumor and disk (Figure 6B,C). The lesion was easily separated from surrounding tissues. When the left condyle with tumor was removed, the mandible, which had deviated to the right, was easily restored and good occlusion was achieved. The tumor was 18 mm in maximum diameter (Figure 7).

**Figure 1:**
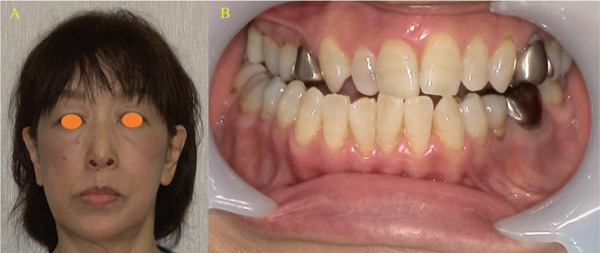
Preoperative facial and oral photographs. (A) Facial photograph: The mandible is deviated to the right. (B) Oral photograph: The mandibular central incisors deviated 8 mm to the right toward the maxillary central incisors.

**Figure 2:**
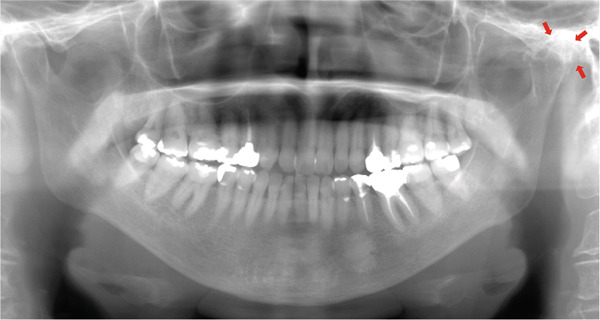
Panoramic radiograph: A bone‐like radio opaque mass is seen posterior to the left mandibular condyle (arrow).

**Figure 3:**
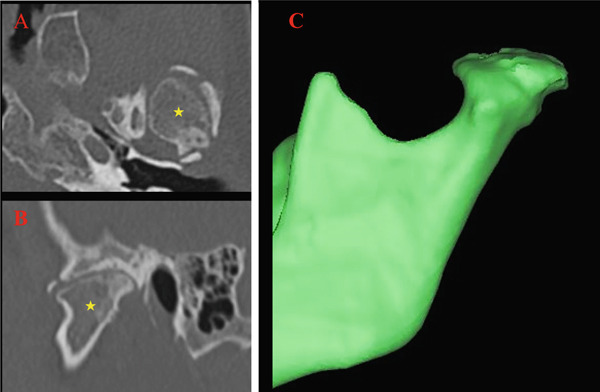
CT images: (A) Axial CT; ★, condyle. (B) Sagittal CT; ★, condyle. (C) The 3D‐CT model demonstrates a well‐defined, bone‐like, opaque mass located around the mandibular condyle.

**Figure 4:**
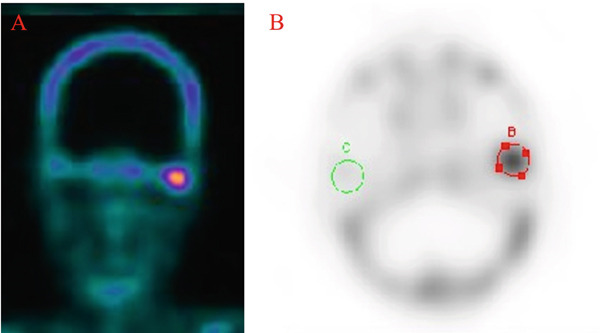
Bone scintigraphy image (^99m^Tc): (A) Bone scintigraphy shows an increase in uptake of radioactive isotope in the left TMJ region. (B) Axial image: Radioactive uptake in the condyle on the affected side is found to be 3.69 times greater than on the unaffected side.

**Figure 5:**
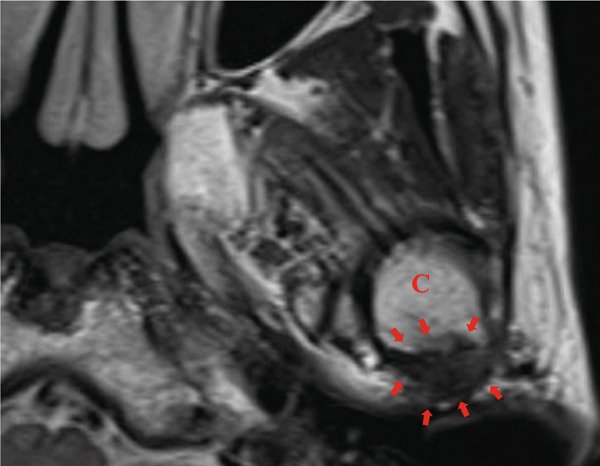
Axial T2‐weighted MRI: MRI reveals a mass lesion (arrows) comprising cortical bone and bone marrow, connected to the cortical bone and bone marrow of the condyle, respectively. C, condyle.

**Figure 6:**
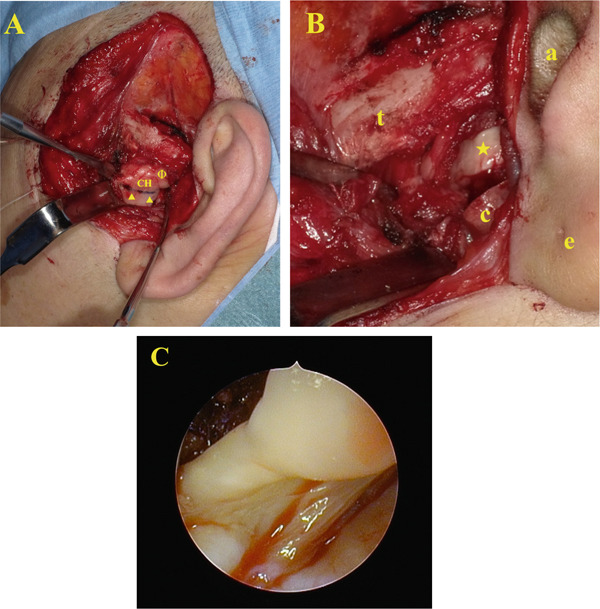
(A, B) Intraoperative photographs and (C) endoscopic image: (A) The tumor and mandibular condyle are clearly defined. The blue line (▲) below the condylar head indicates the osteotomy line. *Φ*, lesion; CH, condylar head. (B) After condylectomy to remove the mandibular condyle and tumor, the articular disk (★) is preserved. t, articular tubercle; c, severed condyle; e, earlobe; a, external auditory meatus. (C) The articular disk appears normal

**Figure 7:**
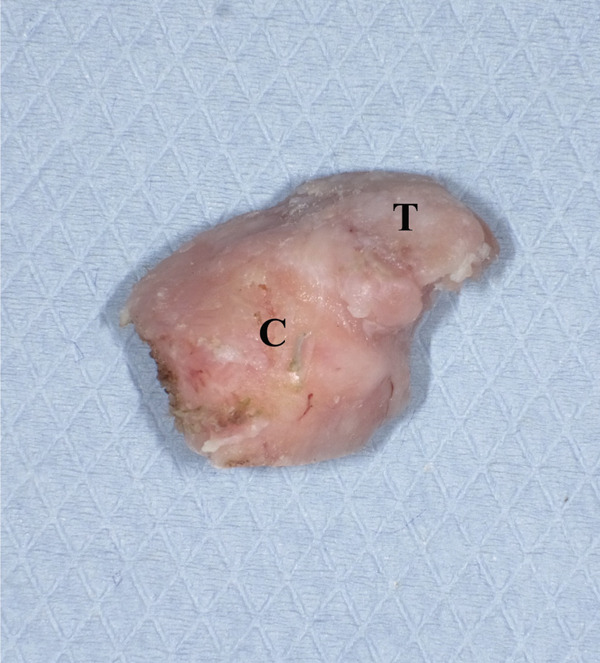
Resected tumor and left condyle. T, tumor; C, condyle.

### 2.1. Pathology

On the cut surface of the extracted mandibular condyle, the mandibular condyle protruded posteriorly and superiorly while maintaining continuity with the cortical bone (Figure 8A). Hematoxylin and eosin (HE) staining demonstrated that the basic structure of the mandibular condyle was generally preserved (Figure 8B). The surface of the mandibular condyle contains fibrous layer, undifferentiated mesenchyme layer, and cartilage layer. Cartilage cap (undifferentiated mesenchyme layer and cartilage layer) shows thin (Figure 8C,D). Partial mild angiogenesis and fibrosis were observed in the bone marrow tissue near the hyaline cartilage. Based on these findings, a definitive diagnosis of mandibular condylar osteochondroma was made. As of 1 year and 6 months postoperatively, both occlusion and facial asymmetry remain substantially improved (Figure 9A,B). No recurrence has been observed.

**Figure 8:**
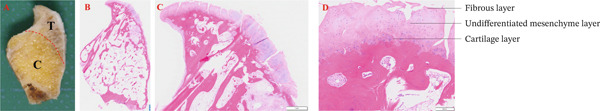
Cut surface of the extracted tissue and hematoxylin and eosin‐stained section: (A) Cut surface of the extracted tissue. C, condyle; T, lesion. The dashed line indicates the boundary between the condyle and the lesion. (B) Histologic section (hematoxylin and eosin staining, ×40). The boundary between the mandibular condyle and lesion is unclear. (C) Histologic section (hematoxylin and eosin staining, scale bar:1 mm). The basic structure of the mandibular condyle is generally preserved. This section shows trabeculae of mature lamellar bone with fatty marrow. (D) Histologic section (hematoxylin and eosin staining, scale bar: 200 *μ*m). The surface of the mandibular condyle contains fibrous layer, undifferentiated mesenchyme layer and cartilage layer. Cartilage cap (undifferentiated mesenchyme layer and cartilage layer) shows thin.

**Figure 9:**
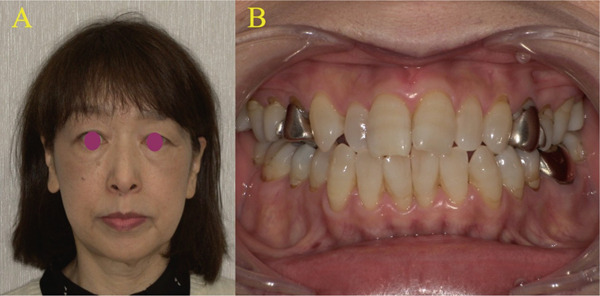
Postoperative facial and oral photographs: (A) Facial photograph: Facial asymmetry has improved. (B) Oral photograph: The midlines of the upper and lower dental arches align.

## 3. Discussion

Pathologies that cause enlargement of the mandibular condyle include tumors and hyperplasia. Tumors arising in the mandibular condyle include osteochondroma, osteoma, osteosarcoma, and osteochondrosarcoma, among others. Imaging modalities such as plain x‐ray, CT, MRI, and bone scintigraphy are used first for differentiation between condylar osteochondroma and other conditions. Distinguishing condylar osteochondroma from CH is particularly difficult and often controversial [9–11]. CH is a rare disease that manifests as nonneoplastic overgrowth of the unilateral mandibular condyle [12]. CH is common and frequently occurs in groups during the growth phase, particularly in adolescence [13]. Females appear more sensitive to CH than males, so sex may be considered a risk factor [14]. Nolte et al. reported a mean age of 20.3 years for this pathology, with a range of 9–54.5 years among 309 cases of CH [15]. As the mandibular condyle grows due to hyperplasia, facial asymmetry and malocclusion occur, similar to osteochondroma. In typical cases, CT findings may enable differentiation between these two diseases [10]. In condylar osteochondroma, multislice CT scans reveal bony lobulated outgrowth with different mineralization patterns in continuation between the cortex and medulla of the condyle with the tumor [16]. The coronal and sagittal CT sections tend to reveal a growth arising from the morphologically normal condyle in contrast to the uniform enlargement of the condylar head, which is characteristic of CH [17]. The morphology of the condyle is clearly changed and uneven, but the length and width of the mandibular ramus on the affected side are not apparently increased [10]. Radiographically, in typical findings of CH, the morphologic enlargement of the condyle and the elongation and thickening of the condylar neck are clearly present. CT scans demonstrate an enlarged and smoothed condyle. Compared with the contralateral side, the length and width of the mandibular ascending and horizontal rami are clearly increased [10]. However, in cases where imaging does not show typical findings, differentiation is often difficult, and considering the possibility of osteochondroma becoming malignant, differentiating between the two pathologies is crucial. Histological examination is very useful for differentiating between osteochondroma and hyperplasia of the condyle. In the normal condyle, a thin layer of cartilage covers the condylar surface. In condylar osteochondroma and hyperplasia, the width of this cartilage layer is thickened, and this thickened cartilage layer is referred to as the “cartilage cap,” a characteristic finding. The cartilage cap is divided into four layers: fibrous layer; undifferentiated mesenchyme layer; cartilage layer including pre‐hypertrophic and hypertrophic chondrocytes; and calcified cartilage layer [9]. In addition, “cartilage islands,” which are observed within the condyle during the growth phase, are present in the inferior cancellous bone. Condylar cartilage in CH shows visibly enlarged mesenchymal cells in the proliferative layer and hypertrophic chondrocytes in the hypertrophic layer, with many cartilage islands observed in the subchondral bone region [10]. On the other hand, condylar osteochondroma shows hyperplastic chondrocytes larger than those in the normal condyle but smaller than those observed in CH, and few cartilage islands are observed in the bone layer [10]. Postoperative histopathological examination has been used for this differentiation [9, 10]. Yu et al. reported that the thicker cartilage cap, higher bone formation rate, and higher proliferating cell nuclear antigen‐positive rate indicated a higher rate of proliferative activity in condylar osteochondroma [9]. Ji et al. described safranin O staining and safranin‐fast green staining as effective staining methods for differentiating between CH and condylar osteochondroma [10]. The same authors suggested that Runx2 findings from immunohistochemical investigations are valuable in differentiating between these two diseases. Runx2, a runt‐domain transcription factor, is an important nuclear factor involved in promoting chondrocyte hypertrophy [18]. The presence of Runx2 is found mainly in the proliferative chondrocyte layer and the hypertrophic chondrocyte layer, and the expression of Runx2 is higher in CH than in condylar osteochondroma [10]. In the present case, the lesion comprised fibrous, cartilaginous, and cancellous bone layers. The chondrocytes were small, and no cartilage islands were observed. CT showed a pedunculated tumor originating from the mandibular condyle. On bone scintigraphy, 3.69 times higher tracer accumulation was observed compared with the contralateral condyle, suggesting an active state [19]. In cases of active CH, cartilage islands should be visible [9]. However, no cartilage islands were observed in our case. Furthermore, when considering age, CH is most commonly seen in younger individuals, as is typical of adolescence. Since the present case began to experience a gradual deviation of the mandible approximately 5 years ago, it is presumed that the onset occurred when the patient was in her late 50s. Although CH may also result from trauma, the present case had no history of head trauma. Based on these findings, osteochondroma was diagnosed. Table 1 summarizes a comparison of the clinical and histological characteristics of osteochondroma of the condyle and CH.

**Table 1:** Clinical and histological findings of osteochondroma and hyperplasia occurring in the condyle.

	Osteochondroma	Hyperplasia
Clinical		
Common age	Adults [6, 20]	Adolescence [13, 15, 21]
Gender difference	Female [6, 17, 20]	Female [13]
Form	Localized nodular, pedunculated [22, 23]	Diffuse enlargement from the condyle head to the neck [24]
Continuity with existing bone (condyle)	Presence: cortical bone and cancellous bone are, respectively, continuous with bone tissue of condyle [3, 25, 26]	Presence: diffuse enlargement of the whole condyle [10, 17]
Histological		
Cartilaginous cap	Thick [9 **]**	Thin [9]
Cartilaginous islands	Few [10]	Many [10]
Hyperplastic chondrocyte	Small [10]	Large [10]

In surgery for osteochondromas, total condylectomy or low condylectomy is performed because the tumor must be completely removed while ensuring a safety margin. The so‐called “low condylectomy” involves an osteotomy line below the head but high in the neck, preserving its entire length and ensuring a radical tumor excision [27]. Total condylectomy significantly reduces the height of the posterior mandible. Depending on the condition following excision, reconstructive procedure of condyle may be necessary; therefore, careful consideration must be given when determining the appropriate course of treatment. To counteract mandibular ramus shortening following low condylectomy, López et al. reported the innovative treatment combining low condylectomy with asymmetric mandibular osteotomies for hemimandibular hyperplasia associated with osteochondroma [28]. On the other hand, in the surgery for CH, local excision or high condylectomy is performed because it only removes the affected area. In high condylectomy, the horizontal osteotomy is usually done 4–5 mm caudal to the edge of the condylar head, leaving a portion of the condylar head intact [29]. Therefore, the appropriate surgical procedures differ between osteochondromas and mandibular CH. However, if the lesion cannot be differentiated between osteochondroma and hyperplasia preoperatively, resection with a safety margin is preferable. In the present case as well, since a definitive diagnosis could not be established preoperatively, low condylectomy was performed.

Currently, tissue biopsy is the only method to obtain a definitive diagnosis preoperatively, but biopsy procedures within the mandibular condyle or articular capsule of the TMJ are not easy and thus not appropriate for differentiation from benign diseases. If postoperative histopathological examination reveals CH, this represents an unavoidable overtreatment. In the future, new knowledge and tests will hopefully enable preoperative differentiation of mandibular condylar osteochondroma and hyperplasia in a noninvasive manner.

## Author Contributions

Conceptualization, data curation, investigation, visualization, and writing: Hisashi Ozaki. Formal analysis and funding acquisition have not been done. Methodology and project administration have not been done. Resources: Hisashi Ozaki, Erika Miyamoto, and Yuichiro Imai. Software has not been done. Supervision: Yoshihiko Yokoe and Yuichiro Imai. Validation has not been done. Writing—review and editing: Hisashi Ozaki, Erika Miyamoto, Hiroki Mori, Yosuke Kobayashi, Yoshihiko Yokoe, and Yuichiro Imai.

## Funding

No funding was received for this manuscript.

## Disclosure

All authors have read and approved the final version of the manuscript. Corresponding author had full access to all of the data in this study and takes complete responsibility for the integrity of the data and the accuracy of the data analysis.

## Ethics Statement

Single anonymized case report with informed consent.

## Consent

Informed consent was obtained from the patient for publication of this case report and any accompanying images.

## Conflicts of Interest

The authors declare no conflicts of interest.

## Supporting information


**Supporting Information** Additional supporting information can be found online in the Supporting Information section. 

## Data Availability

The data that support the findings of this study are available on request from the corresponding author. The data are not publicly available due to privacy or ethical restrictions.
