# Supplementary material for: Condylar Osteochondroma in an Older Adult With Facial Asymmetry: Case Report and Differentiation From Condylar Hyperplasia
Source: Case Rep Dent. 2026 Aug 13;2026:4143126. doi: 10.1155/crid/4143126 (PMC13470025; doi:10.1155/crid/4143126)
Supplement: Supplementary file 1 [file CRID-2026-4143126-s001.pdf]

CARE case report guidelines

CARE Checklist of information to include when writing a case report

(cc) BY-NC-ND

✓

Topic	Item	Checklist item description	Reported on Line
Title	1	The diagnosis or intervention of primary focus followed by the words "case report"	Page 1, line 2
Key Words	2	2 to 5 key words that identify diagnoses or interventions in this case report, including "case report"	Page 2, line 20 to Page 3, line 1
Abstract (no references)	3a	Introduction: What is unique about this case and what does it add to the scientific literature?	Page 2, lines 2-8
	3b	Main symptoms and/or important clinical findings	Page 2. lines 8-9
	3c	The main diagnoses, therapeutic interventions, and outcomes	Page 2. lines 9-15
	3d	Conclusion—What is the main "take-away" lesson(s) from this case?	Page 2. lines 16-18
Introduction	4	One or two paragraphs summarizing why this case is unique (**may include references**)	Page 4. lines 2 to Page 5. line 1
Patient Information	5a	De-identified patient specific information.	Page 5. lines 4-5
	5b	Primary concerns and symptoms of the patient.	Page 5. lines 5-8
	5c	Medical, family, and psycho-social history including relevant genetic information	Page 5. lines 13-14
	5d	Relevant past interventions with outcomes	none
Clinical Findings	6	Describe significant physical examination (PE) and important clinical findings.	Page 5. lines 8 to Page 6. lines 8
Timeline	7	Historical and current information from this episode of care organized as a timeline	none
Diagnostic Assessment	8a	Diagnostic testing (such as PE, laboratory testing, imaging, surveys).	Page 5 lines 8 to Page 6. lines 9
	8b	Diagnostic challenges (such as access to testing, financial, or cultural)	none
	8c	Diagnosis (including other diagnoses considered)	Page 7. lines 11-12
	8d	Prognosis (such as staging in oncology) where applicable	Page 7. lines 12-14
Therapeutic Intervention	9a	Types of therapeutic intervention (such as pharmacologic, surgical, preventive, self-care)	Page 6. lines 11
	9b	Administration of therapeutic intervention (such as dosage, strength, duration)	Page 6. lines 11 to Page 7. lines 3
	9c	Changes in therapeutic intervention (with rationale)	none
Follow-up and Outcomes	10a	Clinician and patient-assessed outcomes (if available)	Page 7. lines 12-14
	10b	Important follow-up diagnostic and other test results	none
	10c	Intervention adherence and tolerability (How was this assessed?)	none
	10d	Adverse and unanticipated events	none
Discussion	11a	A scientific discussion of the strengths AND limitations associated with this case report	Page 7. lines 16 to Page 12. line 8
	11b	Discussion of the relevant medical literature **with references**.	Page 8. lines 2 to Page 11. line 2
	11c	The scientific rationale for any conclusions (including assessment of possible causes)	Page 11. lines 3 to Page 12. line 1
	11d	The primary "take-away" lessons of this case report (without references) in a one paragraph conclusion	Page 12. lines 2-8
Patient Perspective	12	The patient should share their perspective in one to two paragraphs on the treatment(s) they received	none
Informed Consent	13	Did the patient give informed consent? Please provide if requested	Yes ☑ No ☐
